# The impact of different sprayable surfaces on the effectiveness of indoor residual spraying using a micro encapsulated formulation of lambda-cyhalothrin against *Anopheles gambiae s.s.*

**DOI:** 10.1186/s13071-015-0795-4

**Published:** 2015-04-03

**Authors:** Joshua Mutagahywa, Jasper N Ijumba, Harish B Pratap, Fabrizio Molteni, Frances E Mugarula, Stephen M Magesa, Mahdi M Ramsan, Jessica M Kafuko, Elias C Nyanza, Osia Mwaipape, Juma G Rutta, Charles D Mwalimu, Isaiah Ndong, Richard Reithinger, Narjis G Thawer, Jeremiah M Ngondi

**Affiliations:** RTI International, Dar es salaam, Tanzania; Department of Zoology and Wildlife Conservation College of Natural and Applied Sciences, University of Dar es salaam, Dar es salaam, Tanzania; Nelson Mandela African Institute of Science and Technology, Arusha, Tanzania; Swiss Tropical and Public Health Institute, Dar es salaam, Tanzania; National Malaria Control Program, Ministry of health and Social Welfare, Dar es salaam, Tanzania; Sengerema Health Institute, Sengerema, Tanzania; United States Agency for International Development, Abuja, Nigeria; School of Public Health, Catholic University of Health and Allied Sciences, Mwanza, Tanzania; RTI International, North Carolina, USA; RTI International, Washington, DC USA

**Keywords:** Indoor residual spraying, Lambda-cyhalothrin, Wall surfaces, *Anopheles gambiae ss*, Mainland Tanzania, Zanzibar

## Abstract

**Background:**

The type of sprayable surface impacts on residual efficacy of insecticide used in indoor residual spraying (IRS). However, there is limited data on common types of wall surfaces sprayed in Zanzibar and mainland Tanzania where IRS began in 2006 and 2007 respectively. The study investigated residual efficacy of micro-encapsulated lambda-cyhalothrin sprayed on common surfaces of human dwellings and domestic animal shelters in Zanzibar and mainland Tanzania.

**Methods:**

An experimental hut was constructed with different types of materials simulating common sprayable surfaces in Zanzibar and mainland Tanzania. Surfaces included cement plastered wall, mud-daub, white-wash, wood, palm-thatch, galvanized iron-sheets, burnt-bricks, limestone and oil-paint. The World Health Organization (WHO) procedure for IRS was used to spray lambda-cyhalothrin on surfaces at the dose of 20–25 mg/m^2^. Residual efficacy of insecticide was monitored through cone bioassay using laboratory-reared mosquitoes; Kisumu strain (R–70) of *Anopheles gambiae ss*. Cone bioassay was done every fortnight for a period of 152 days. The WHO Pesticide Evaluation Scheme (WHOPES) threshold (80% mortality) was used as cut-off point for acceptable residual efficacy.

**Results:**

A total of 5,800 mosquitoes were subjected to contact cone bioassay to test residual efficacy of lambda-cyhalothrin. There was a statistically significant variation in residual efficacy between the different types of wall surfaces (r = 0.24; p < 0.001). Residual efficacy decreased with increasing pH of the substrate (r = −0.5; p < 0.001). Based on WHOPES standards, shorter residual efficacy (42-56 days) was found in wall substrates made of cement, limestone, mud-daub, oil paint and white wash. Burnt bricks retained the residual efficacy up to 134 days while galvanized iron sheets, palm thatch and wood retained the recommended residual efficacy beyond 152 days.

**Conclusion:**

The study revealed a wide variation in residual efficacy of micro encapsulated formulation of lambda-cyhalothrin across the different types of wall surfaces studied. In areas where malaria transmission is bimodal and wall surfaces with short residual efficacy comprise > 20% of sprayable structures, two rounds of IRS using lambda-cyhalothrin should be considered. Further studies are required to investigate the impact of sprayable surfaces on residual efficacy of other insecticides commonly used for IRS in Zanzibar and mainland Tanzania.

## Background

Over the last decade, many countries including Tanzania have strengthened malaria control using a combination of both insecticide treated mosquito nets (ITNs) and indoor residual spraying (IRS) [1]. IRS remains a powerful vector control tool for reducing and interrupting malaria transmission in malaria endemic areas. In Africa, more than 77 million people benefited from the intervention in 2011 and the number continue to increase [2]. IRS is one of the core interventions to control malaria vectors both in Zanzibar and mainland Tanzania [3,4]. Between 2006 and 2012, three to six blanket rounds of IRS were implemented, covering 85 – 90% of all eligible structures in Zanzibar and three regions (Kagera, Mwanza and Mara) of mainland Tanzania [5]. It was during this period that the areas where IRS was implemented registered dramatic decline of malaria prevalence from 25% to less than 1% in Zanzibar, 41% to 8% in Kagera, 31.4% to 19% in Mwanza and 30.3% to 25% in Mara [6]. This decline was also attributed to the implementation of long lasting insecticidal nets (LLIN), and malaria case management with malaria rapid diagnostic tests (mRDT) and artemisinine combination therapy (ACTs).

There is strong evidence that supports the efficacy and effectiveness of IRS in malaria control in countries where it was implemented appropriately [2,7]. The intervention has potential to significantly reduce malaria morbidity and mortality and accelerate progress towards global and national malaria control targets [8,9]. Lambda-cyhalothrin (both wettable and micro encapsulated formulations) has been widely used for IRS in Zanzibar and mainland Tanzania. The choice of lambda-cyhalothrin was based on the good profile of the insecticide and baseline studies that supported high susceptibility of *An. Gambiae s.l.* [10]. The other criteria used to select lambda-cyhalothrin were its comparatively low cost compared to other insecticides. In addition, despite its high toxicity to bees and fish, it has low mammalian toxicity [11].

Residual efficacy of an insecticide on the sprayed surfaces is an important factor that influences the effectiveness of IRS [12]. The insecticide should be sufficiently stable to maintain biological efficacy on treated surfaces over time in order to minimize the number of spraying cycles required to cover the targeted malaria transmission seasons [2]. An insecticide for IRS is considered to have adequate residual efficacy when mortality of the exposed mosquitoes is ≥ 80% at 24 hours post-exposure [13]. Factors influencing efficacy include: mosquito susceptibility to insecticide; mosquito behavior (endophilic and endophagic); type of sprayable surfaces; quality of IRS; and community acceptance [14,15]. Other factors have been reported to contribute to the residual efficacy of an insecticide, such as: the type of insecticide; formulation; applied dose; physical and chemical properties of the sprayed surfaces; and weather conditions [16-18].

Varying residual efficacy of lambda-cyhalothrin (ICON® 10CS) has been reported ranging between 2 – 7 months on various surfaces [19-23]. Anecdotal entomological findings suggested that residual efficacy of lambda-cyhalothrin varied with different types of sprayable wall surfaces [5]. In Zanzibar and mainland Tanzania, IRS monitoring data revealed various types of sprayable surfaces. However, there are limited studies on the influence of different wall substrate on the residual efficacy of insecticides commonly used for IRS in Zanzibar and mainland Tanzania. The study investigated residual efficacy of micro-encapsulated lambda-cyhalothrin sprayed on common surfaces of human dwellings and domestic animal shelters in Zanzibar and Mainland Tanzania.

## Methods

### Study site

**Figure 1:**
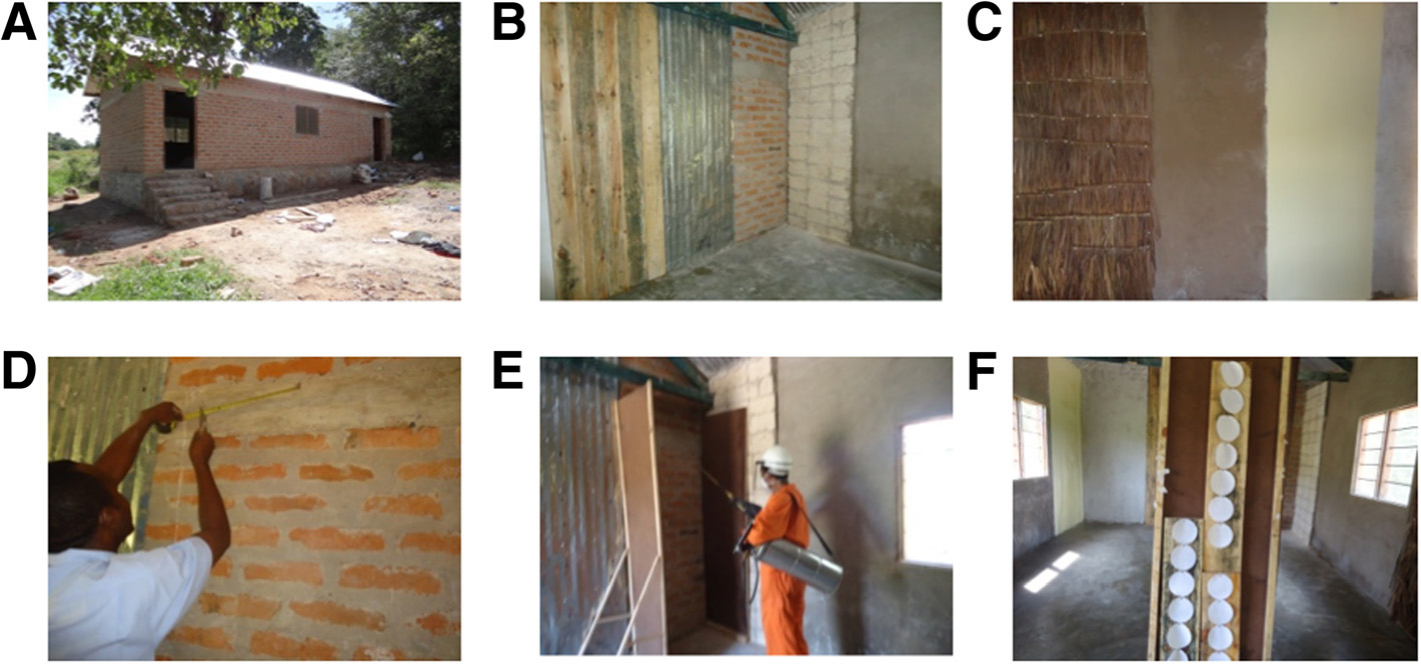
**Design of the experimental hut and procedure used for spraying. A**. An experimental hut with two rooms was constructed assembling nine different surfaces. **B**. Surfaces of wood, iron sheet, burnt bricks, limestone blocks and cement plastered walls are shown. **C**. Surfaces of palm thatch, mud daub and oil paint are shown. **D**. The substrates were fixed on the walls of the hut and demarcated to guide the spraying movement **E**. Spraying on the tested surfaces was done using side edges to avoid overlap effect. **F**. Whatman paper was hinged on wood after being sprayed on tested surfaces.

The study was conducted at National Institute for Medical Research (NIMR) Mwanza center in Northern Tanzania. An experimental hut was constructed assembling nine different surfaces which are commonly sprayed structures of human dwellings and domestic animal shelters both, in Zanzibar and mainland Tanzania (Figure 1).

### Study design

In the experimental study design, treatment and control groups were used. The experimental hut was divided into two rooms and each room comprised 9 substrates plastered in the inside walls (Figure 1A-C). One room contained treated units that were replicated twice and the other room contained one surface of each study unit as the control. The substrates were fixed on the walls of the hut and demarcated to guide the spraying movement (Figure 1D). Side edges were used during spraying to avoid overlap effect (Figure 1E). The treated room also comprised of Whatman paper hinged on wood after being sprayed on tested surfaces (Figure 1F). Units of the study were fabricated surfaces (1.0 m wide and 2.5 m high) of limestone blocks, burnt bricks, white wash, galvanized iron sheet, cement plastered wall, mud daub, wood, palm thatch and oil paint.

### Determination of substrates pH

Before spraying, the study substrates were tested for pH which was assumed to be one of the possible causes for accelerating insecticide decay. The corrugated iron sheet was not tested due to methodological limitations. The measurements were done using a hand held pH meter (HK 3C 8818) according to methods developed by Campbell and Bryant [24]. Using a scalpel, the substrate was scraped off from wall surfaces of white wash, burnt bricks, limestone blocks, cement blocks and mud daub. The materials obtained were sieved using a domestic sifter used to sieve flour. For the wood and palm thatch, the experiment used powder obtained while shredding the materials using a hand saw. Each sample was replicated twice. The substrate and distilled water were mixed in a jar with a ratio of 1:5 weights by volume (20 g: 100 ml). The mixture was shaken for five minutes to allow leaching of ions and then left to settle for ten minutes after which pH was measured.

### Spraying of the surfaces with lambda-cyhalothrin

The surfaces were sprayed with lambda-cyhalothrin (ICON 10CS®). Before spraying, the surfaces were marked with three lines, one in the middle where the nozzle was pointing from 45 cm off the wall while spraying. The other two lines were 27.5 cm from the middle on either side. The marking identified the area of optimal concentration of the swathe (55 cm), avoiding 10 cm in each side that receives fewer doses [14]. The spraying was done to attain the dose of 20 – 25 mg/m^2^ which is within the recommended range for malaria vector control [25]. The spraying used Hudson® hand compression sprayers with a flat nozzle (SS 8002), which is recommended for the application of public health insecticides [26]. Pressure in the pump ranged between 1.8 – 4 kg/m^2^. Distance from the sprayed wall was maintained at 45 cm, while up and down movement was at 2.2 seconds per meter.

### Insecticide residue analysis

Prior to spraying, three pieces of filter papers (Whatman® paper No 1) were pinned on each surface: one on the upper (130 – 250 cm upper); the second in the middle (125 cm middle); and the third on the lower segment (1 – 20 cm). A total of 54 filter paper pieces were pinned (3 pieces per wall × 2 replicates × 9 surfaces). After spraying, 18 pieces of filter paper were randomly selected for laboratory residual analysis to determine the amount of insecticide that was deposited on the walls through spraying procedure. Only 1/3 of the strips (18/54) were randomly selected and analyzed for the amount of insecticide available, due to high cost of analyzing filter paper strips (40$ per piece). The remaining 36 were hinged on the wood surface and kept in the experimental hut for cone bioassay to expose them to the same environmental conditions as the tested substrates. The natural decay of insecticide was determined on the filter papers since it exerts minimum physical and chemical influence on the insecticide and was thus used a positive reference control [27]. Capillary Gas Chromatograph (Agilent 7890A Gas Chromatography Mass Spectrophotometer) was used to determine residues on the sprayed filter papers.

### Mosquito rearing

Mosquito rearing was routinely undertaken by a qualified insectary attendant at NIMR, Mwanza. The purpose of rearing mosquitoes was to raise a colony of mosquitoes to cope with the demand of cone bioassays, synchronize their age structure so that experiments would be done with mosquitoes of same age to ensure reliability and reproducibility of data [28].

### Cone bioassay

A laboratory-reared Kisimu strain (R-70) of *An. gambiae s.s* was subjected to contact bioassays on different sprayed surfaces. Two-day old, unfed *An. gambiae s.s* females were siphoned out of a cage using mouth-operated aspirators and gently blown into each cone bioassay fixed on the test material with the aid of masking tape and/or elastic bands [29]. Each cone had a maximum of 20 female mosquitoes. After exposing mosquitoes for 30 minutes, they were siphoned out of the cone and blown into a holding paper cup, fed on 10% glucose and kept at room temperature. Mortality was scored after 1 hour and 24 hours post exposure. Any mortality occurring within 1 hour post exposure was scored as knock-down [28]. Cone bioassay was done every fortnight throughout 152 days using 20 mosquitoes per cone.

### Data analysis

Two sets of data (pH of substrates and mosquito mortality) were analyzed using SPSS® (Statistical Package for Social Science version 18) and Microsoft Excel®. Regression analysis model was used to predict the mortality rate of the mosquitoes from day 1 to day 152 for each substrate. Analysis of Variance (ANOVA) was used to test the statistical significance of the differences in mean mosquito mortality rate. Multiple analyses of variance (MANOVA) was used to perform multiple comparisons for the tested surfaces. Whatman® paper was used as a reference substrate against other substrates as it was considered to have very minimal physical and chemical influence of efficacy of lambda-cyhalothrin.

### Ethical consideration

The study protocol was approved by the institutional review committee of University of Dar es Salaam. The study did not involve any human subjects or animals.

## Results

### Concentration of lambda-cyhalothrin

Table 1, shows the estimated dose of insecticide that was deposited on the wall substrates based on quantification from 18 randomly selected filter papers that were pinned on the substrates prior to spraying. The results indicate that, the concentration of insecticide sprayed on the wall substrates was within the recommended range for IRS of 20 – 25 mg/m^2^.

**Table 1:** **Lambda-cyhalothrin concentration levels on the sprayed substrates**

**Substrate**	Sample location*	**mg/l**	**mg/m** ^**2**^	**Substrate**	Sample location*	**mg/l**	**mg/m** ^**2**^
Cement blocks	B	2.22E-02	22	Limestone blocks	F	2.48E-02	25
Cement blocks	E	2.38E-02	24	Limestone blocks	A	2.18E-02	22
Cement blocks	F	2.23E-02	22	Iron sheet	C	2.20E-02	22
Mud daub	A	2.30E-02	23	Burnt bricks	B	2.19E-02	22
Mud daub	E	2.47E-02	25	Palm thatch	A	2.51E-02	25
Wood	B	2.15E-02	22	Palm thatch	D	2.23E-02	22
Wood	D	2.39E-02	24	White wash	C	2.32E-02	23
Oil paint	A	2.00E-02	20	White wash	A	2.36E-02	24
Oil paint	D	2.01E-02	20	White wash	B	2.17E-02	22

*The sample location refers to the level where the whatman paper was located. A = Upper, B = Middle, C = Lower levels of the 1^st^ replicate; D = Upper, E = Middle, F = Lower levels of the 2^nd^ replicate.

### Residual efficacy of lambda-cyhalothrin

The residual efficacy of lambda-cyhalothrin on tested wall substrates was monitored for a period of 152 days. There was variation in residual efficacy between the different types of wall surfaces (r = 0.24; p < 0.001).

### Decay of residual efficacy

**Figure 2:**
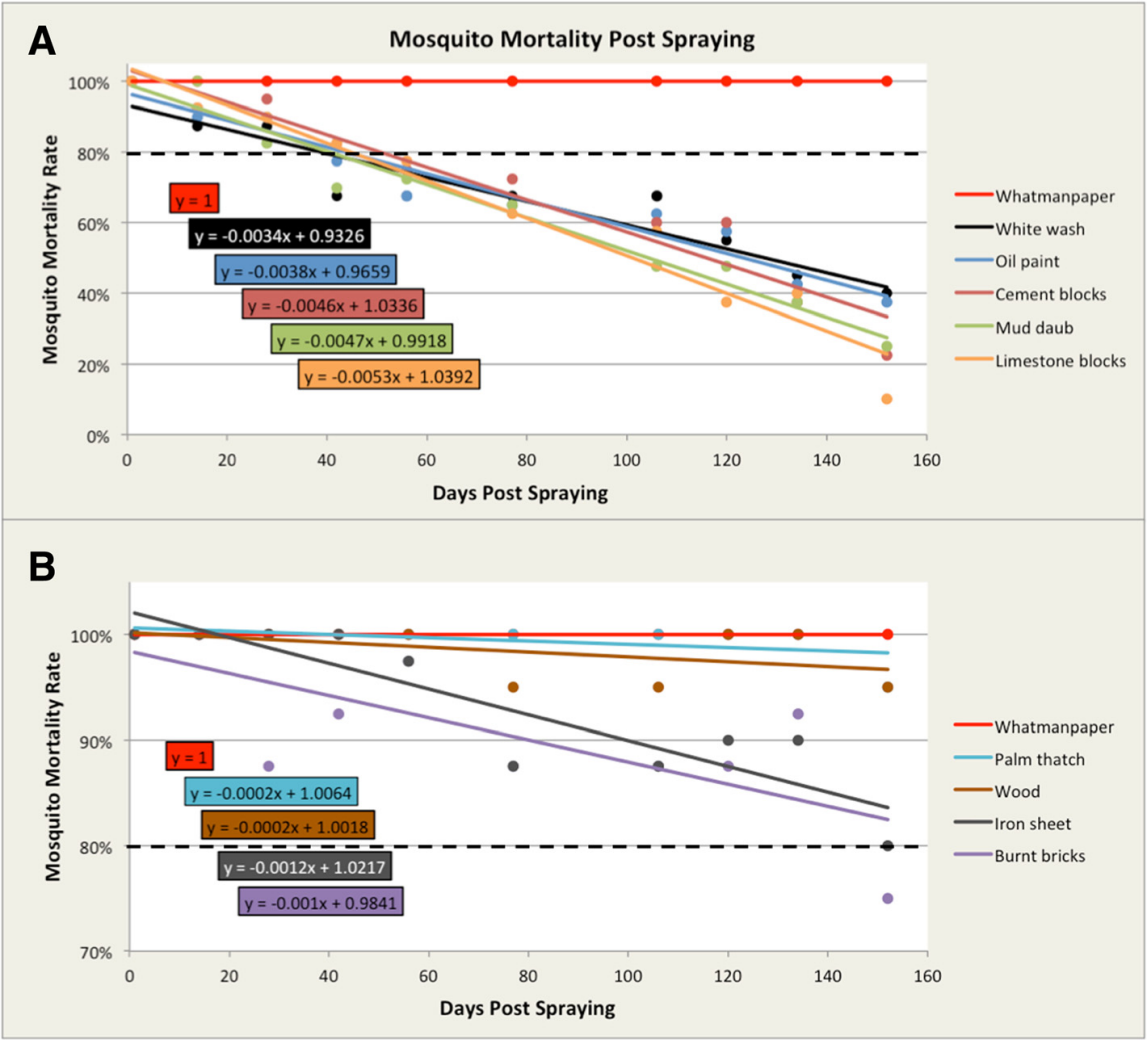
**Mosquito mortality rate on wall substrates (A) that did not meet the WHO standards during the study period and (B) that met the WHO standards throughout the study period.** Whatman paper was used as a reference point. The dashed line represents the WHO threshold of 80% mosquito mortality rate.

Different patterns of residual efficacy decay were observed among the wall substrates tested as shown in Figures 2 and Table 2. The results are presented using regression curves that show the rate of insecticide decay versus number of days post spraying (Figure 2). Based on World Health Organization Pesticide Evaluation Scheme (WHOPES) standards, shorter residual efficacy (42–56 days) was found in wall substrates made of cement, limestone, mud-daub, oil paint and white wash (Figure 2A). However, burnt bricks retained the residual efficacy up to 134 days while walls made of iron sheets, palm thatch and wood, retained the recommended efficacy of ≥80% mortality beyond 152 days (Figure 2B). For the untreated control groups, the mosquito mortality was 0% for all tested substrates.

**Table 2:** **Mortality rate (%) of** ***Anopheles gambiae s.s.*** **exposed to different surfaces sprayed with a micro-encapsulated formulation of lambda cyhalothrin (10CS)**

**Substrate**	**Days post spraying**									
	**1**	**14**	**28**	**42**	**56**	**77**	**106**	**120**	**134**	**152**
Burnt bricks	100%	100%	88.0%	93.0%	98.0%	88.0%	88.0%	88.0%	93.0%	75.0%
Cement blocks	100%	93.0%	95.0%	83.0%	75.0%	73.0%	60.0%	60.0%	38.0%	23.0%
Iron sheet	100%	100%	100%	100%	98.0%	88.0%	88.0%	90.0%	90.0%	80.0%
Limestone blocks	100%	93.0%	90.0%	83.0%	78.0%	63.0%	58.0%	38.0%	40.0%	10.0%
Mud daub	100%	100%	83.0%	70.0%	73.0%	65.0%	48.0%	48.0%	38.0%	25.0%
Oil paint	100%	90.0%	90.0%	78.0%	68.0%	65.0%	63.0%	58.0%	43.0%	38.0%
Palm thatch	100%	100%	100%	100%	100%	100%	100%	100%	100%	95.0%
White wash	100%	88.0%	88.0%	68.0%	68.0%	68.0%	68.0%	55.0%	45.0%	40.0%
Wood	100%	100%	100%	100%	100%	95.0%	95.0%	100%	100%	95.0%
Whatmanpaper (control)	100%	100%	100%	100%	100%	100%	100%	100%	100%	100%

### Association of mosquito mortality and pH of surfaces

Table 3 shows the results of pH values on each tested surface except galvanized iron sheet. The pH of the tested surfaces ranged between 5.9 – 11. Increasing pH was associated with decreasing mean mosquito mortality rate (r = −0.5; p <0.001).

**Table 3:** **Relationship between mean mosquito mortality rate and pH of the tested surfaces**

**Substrate type**	**Mean pH**	No. of mosquitoes exposed over 10 time points*	**Mean mortality**	**Std. deviation**
Burnt Bricks	7.64	400	18	±1.631
Cement Blocks	10.3	400	14	±4.989
Oil Paint	10.28	400	14	±4.124
Mud daub	7.71	400	13	±3.829
White wash	11.5	400	14	±3.771
Palm thatch	5.95	400	19	±1.338
Limestone Blocks	10.21	400	13	±5.675
Wood	7.3	400	20	±0.733
Whatman® paper (standard)	7	400	20	±0.0

*2 replicates of 20 mosquitoes per time period.

## Discussion

The study investigated residual efficacy of micro encapsulated formulation of lambda-cyhalothrin (at the dose of 20 – 25 mg/m^2^) on wall substrates comprising mud daub, limestone blocks, cement blocks, white wash and oil paint. Based on WHOPES recommendations, an ideal insecticide should have a minimum effect of ≥ 80% mosquito mortality at 24 hours post exposure on the sprayed surface for 30 minutes [13]. This study revealed that lambda-cyhalothrin residual efficacy on mud daub, limestone blocks, cement blocks, white wash and oil painted surface was below the threshold of ≥80% after 42–56 days post spraying. These findings are in line with previous studies that reported that limestone blocks and white wash, had shorter residual effect (60–120 days) with pyrethroids while wood, ceramic and thatched walls indicated longer residual effects (≥180 days) [30,31].

A key strength of our study design was that we assembled most of the commonly used materials for constructing walls in mainland Tanzania and Zanzibar, including limestone blocks that were transported from Pemba to Mwanza. However, the study had a number of potential limitations. It was not possible to monitor residual efficacy beyond 152 days; therefore, the complete period of effective residual efficacy of lambda-cyhalothrin on different wall substrates could not be established. Our study design used 20 mosquitos per cone during the bioassay. This number is higher than that used in similar studies. It has been argued that too many mosquitoes per cone may increase mortality. However, we do not expect this to have influenced mortality since a similar number of mosquitoes were used for the controls and mortality was 0 for all controls.

Our findings provide important evidence for programmatic decision making. Lambda-cyhalothrin (wettable powder [WP] and micro encapsulated [CS]) has been widely used for IRS to control malaria vectors in mainland Tanzania and Zanzibar. For these IRS operations, the residual effect of the insecticide was largely considered to be 6–9 months as per the manufacturers recommendations [4]. However, this perception and practice was not ideal, since findings of the study revealed a much shorter residual effect of 2–3 months on most surfaces that are common in mainland Tanzania and Zanzibar. These findings suggest that similar residual efficacy studies should be recommended to investigate if insecticides for IRS provide sufficient protection, especially when new insecticides or formulations are introduced.

In mainland Tanzania (Kagera, Mwanza and Mara regions), mud walls (81%) and cement brick walls (17%) constitute a large proportion of sprayable surfaces [5]. Based on our findings, implementation of two rounds of IRS with micro encapsulated formulation of lambda-cyhalothrin (ICON 10CS) should be considered where mud daub, cement blocks, limestone blocks, white-wash and oil painted walls comprise a significant proportion, and where malaria transmission is bimodal. Furthermore, since pH has significant influence on the residual efficacy of lambda-cyhalothrin, assessment done prior to IRS should measure pH of a sample of substrates of targeted house structures. These data can help to predict the impact of pH on IRS using lambda-cyhalothrin. Further research on the influence of common sprayable substrates on the effectiveness of IRS using other insecticides registered for use such as carbamates, organophosphates and organochlorines should be considered.

## Conclusion

The study revealed a wide variation in residual efficacy of micro encapsulated formulation of lambda-cyhalothrin across the different types of wall surfaces studied. In areas where malaria transmission is bimodal and wall surfaces of short residual efficacy comprise >20% of sprayable structures, two rounds of IRS using lambda-cyhalothrin should be considered. Further studies are required to investigate the impact of sprayable surfaces on residual efficacy of other insecticides commonly used for IRS in Zanzibar and mainland Tanzania.

## Abbreviations

ACT: Artemisinine combination therapy
LLIN: Long lasting insecticide nets
IRS: Indoor residual spraying
ITN: Insecticide treated mosquito nets
WP: Wettable powder
CS: Micro-encapsulated
ANOVA: Analysis of variance
MANOVA: Multiple analysis of variance
WHO: World Health Organization
WHOPES: World Health Organization pesticide evaluation scheme
